# A case report: Severe disseminated infection caused by *Strongyloides stercoralis* in an immunocompromised patient by metagenomic next-generation sequencing

**DOI:** 10.3389/fcimb.2023.1082412

**Published:** 2023-04-14

**Authors:** Qinfu Xu, Xiaotong Xi, Dan Feng, Qian Sang, Yanbing Sheng, Ran Ding, Aiguo Xu

**Affiliations:** ^1^ Department of Respiratory, The First Affiliated Hospital of Zhengzhou University, Zhengzhou, Henan, China; ^2^ The State Key Laboratory of Translational Medicine and Innovative Drug Development, Nanjing, China; ^3^ The Medical Department, Nanjing Simcere Medical Laboratory Science Co., Ltd, Nanjing, China

**Keywords:** *Strongyloides stercoralis*, disseminated infection, immunocompromised, metagenomic next-generation sequencing, case report

## Abstract

**Background:**

*Strongyloides stercoralis* (*S. stercoralis*) is a nematode that is widely distributed in the tropical and subtropical regions of the world and which can cause severe disseminated infection in immunocompromised individuals. However, strongyloidiasis, the disease caused by *S. stercoralis*, is difficult to diagnose because of its non-specific clinical presentation and the inadequate performance of conventional diagnostic methods.

**Case description:**

We report the case of a 75-year-old male patient with severe disseminated infection caused by *S. stercoralis*. The patient had a medical history of seasonal bronchitis and, as a consequence, had taken prednisone for many years. Initial clinical tests failed to detect any pathogens, but metagenomic next-generation sequencing (mNGS) resulted in the identification of *S. stercoralis* in the patient’s bronchoalveolar lavage fluid (BALF) and blood. Subsequently, routine testing repeatedly detected nematode larvae in the patient’s stool and sputum. Through a combination of mNGS results and clinical symptoms, the patient was finally diagnosed with severe disseminated infection caused by *S. stercoralis*.

**Conclusion:**

The clinical manifestations of disease caused by infection with *S. stercoralis* are not specific; therefore, early and accurate diagnosis is very important. mNGS can detect *S. stercoralis* even when it is present at only a low level. This case report supports the notion that mNGS is a valuable tool in the diagnosis of severe disseminated infections caused by *S. stercoralis* in immunocompromised patients.

## 1 Introduction

Strongyloidiasis, a systemic disease caused by *Strongyloides stercoralis* (*S. stercoralis*), is endemic to tropical and subtropical regions (Levenhagen and Costa-Cruz, 2014). *S. stercoralis* infection was first reported in French soldiers working in Vietnam who had severe diarrhea, and for many years the disease caused by this organism was known as “Cochin-China diarrhea” (Siddiqui and Berk, 2001). Humans, the main hosts of adult parasites in the parasitic generation, become infected with *S. stercoralis* mainly through skin or mucosal contact with contaminated soil or water, with the main invasion sites being the skin and the respiratory and digestive tracts (Kassalik and Mönkemüller, 2011). Human infection with *S. stercoralis* presents as one of three types: autoinfection, hyperinfection, or disseminated infection. Autoinfection is a mostly asymptomatic process that enables the parasite to survive indefinitely in the human host. Hyperinfection refers to a process of intense autoinfection, during which third-stage larvae can be detected in fresh stool. In the case of disseminated infection, larvae can be found in multiple tissues and body fluids, which can lead to diffuse tissue damage and even death (Farthing et al., 2020). Here, we report a case of severe disseminated infection caused by *S. stercoralis*, diagnosed through a combination of clinical tests and the metagenomic next-generation sequencing (mNGS) of different types of sample [i.e., stool, sputum, blood, and bronchoalveolar lavage fluid (BALF)].

## 2 Case presentation

A 75-year-old male security guard was transferred to his local hospital in early April 2022 because he had been experiencing dyspnea and fever for over half a month. While in hospital, abdominal distension, abdominal pain, no exhaust, and normal defecation were observed. Abdominal digital radiography (DR) and lung computed tomography (CT) scans revealed, respectively, the presence of intestinal obstruction and chronic inflammation in the lungs. The patient was treated with empiric antimicrobial therapy, nebulization, glucocorticoids, enemas, and other supportive treatments (the specific treatment program is unknown), but their therapeutic effect was unsatisfactory. The patient was admitted to the Department of Emergency Medicine at the First Affiliated Hospital of Zhengzhou University, Zhengzhou, People’s Republic of China, on 22 April to receive further treatment for their fever, chest oppression, chronic obstructive pulmonary disease, and abdominal wall hernia, and, after losing consciousness the following day, was transferred to the hospital’s intensive care unit (ICU).

The patient had a history of bronchitis over many years, with seasonal attacks, and self-reported orally administering prednisone and aminophylline. Following a car accident 20 years prior, the patient underwent intestinal anastomosis, leading to the development of an abdominal incisional hernia. He had no family history of bronchitis, had no travel history, and had not been in contact with infected animals. A physical examination revealed that the patient had a symmetrical barrel chest, with widened intercostal spaces, percussive sounds, and scattered wheezing in both lungs. The results of a routine blood test showed that the patient had low potassium (2.26 mmol/L), sodium (121.10 mmol/L), chlorine (84.7 mmol/L), calcium (0.76 mmol/L) levels, a high white blood cell count (WBC; 12.57 × 10^9^/L) and neutrophil count (11.82 × 10^9^/L), and a normal eosinophil percentage (0.9%). A fecal occult blood immunoassay was weakly positive. The levels of C-reactive proteins (105.87 mg/L) and procalcitonin (0.177 ng/mL) were high, and the parasite protozoa test was negative. Cranial CT revealed the presence of a lacunar cerebral infarction in the lateral basal ganglia, while chest CT revealed multiple nodular, lamella-like, and cable-like high-density shadows with unclear boundaries in both lungs, but particularly in the right lung. Bilateral pleural effusion, bilateral pleural hypertrophy, and a chest radiograph showed the presence of fused slices. The patient was tested for the presence of syphilis and hepatitis B antibodies, and underwent polymerase chain reaction (PCR) tests for respiratory viruses and *Mycobacterium tuberculosis.* All test results were negative.

**Figure 1:**
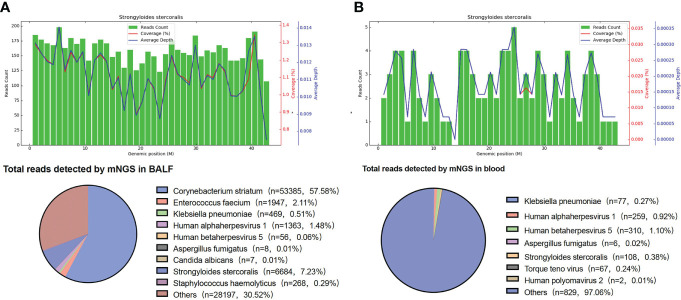
mNGS results. **(A)** *S. stercoralis* detected in BALF by mNGS. The upper panel is *S. stercoralis* reads aligned to its genome. There were 6,684 *S. stercoralis*-specific reads, which accounted for 1.126% of all microbial reads. **(B)** *S. stercoralis* detected in blood by mNGS. The coverage of *S. stercoralis* in blood detected by mNGS is 0.018%. BALF, bronchoalveolar lavage fluid; mNGS, metagenomic next-generation sequencing.

Consideration of the above test results, together with the practitioner’s clinical experience, resulted in the hypothesis that the patient’s condition and presentations were caused by a community-acquired lung infection. Accordingly, the patient was empirically treated with cefpazone and sulbactam (3.0 g, q8h), levofloxacin (0.5 g, qd), and methylprednisolone (40 mg, qd). However, no pathogens were detected after this treatment, indicating its ineffectiveness. On 26 April, BALF samples were sent for further analysis by mNGS in PathogenSeq. DNA was extracted using Tiangen kits in accordance with the manufacturer’s instructions. The DNA library was constructed using a Hieff NGS^®^ OnePotTM II DNA Library Prep Kit. The qualified library was sequenced on the NextSeq 550 platform. After sequencing, adapters and low-quality sequences were removed, and human DNA was filtered by mapping the human reference database. The remaining sequences were aligned to the microbial genome database (NCBI; https://www.ncbi.nlm.nih.gov/genome) to identify potential pathogens. *Corynebacterium striatum* (53,385 specific reads), *Enterococcus faecium* (1,947 specific reads), *Klebsiella pneumoniae* (463 specific reads), human alphaherpesvirus 1 (1, 363 specific reads), human betaherpesvirus 5 (56 specific reads), *Aspergillus fumigatus* (eight specific reads), *Candida albicans* (seven specific reads), and *S. stercoralis* (6,684 specific reads) were identified as the potential infection-causing pathogens. However, as the patient’s home was a considerable distance from any area where *S. stercoralis* is endemic or previous infections had been reported, the chief doctor, in an effort to definitively confirm or dismiss the hypothesis of community-acquired infection, on 28 April ordered that further blood samples be sent for mNGS testing. Routine stool testing was also carried out. *Klebsiella pneumoniae* (77 specific reads), *Aspergillus fumigatus* (six specific reads), human betaherpesvirus 5 (310 specific reads), human alphaherpesvirus 1 (259 specific reads), torque teno virus (67 specific reads), and *S. stercoralis* (108 specific reads) were detected as the potential pathogens (**Figure 1**). **Table 1** lists the pathogens and reads detected by mNGS in both BALF and blood samples.

**Table 1:** The pathogens and reads detected by mNGS in BALF and blood samples.

Date	26/04/2022	27/04/2022
Sample type	BALF	Blood
Pathogen	reads	reads
*Corynebacterium striatum*	55,385	–
*Enterococcus faecium*	1,947	–
*Klebsiella pneumoniae*	469	77
*Aspergillus fumigatus*	8	6
*Candida albicans*	7	–
Human alphaherpesvirus 1	1,363	259
Human betaherpesvirus 5	56	310
*S. stercoralis*	6,684	108
Torque teno virus	–	67

BALF, bronchoalveolar lavage fluid; mNGS, metagenomic next-generation sequencing.

**Figure 2:**
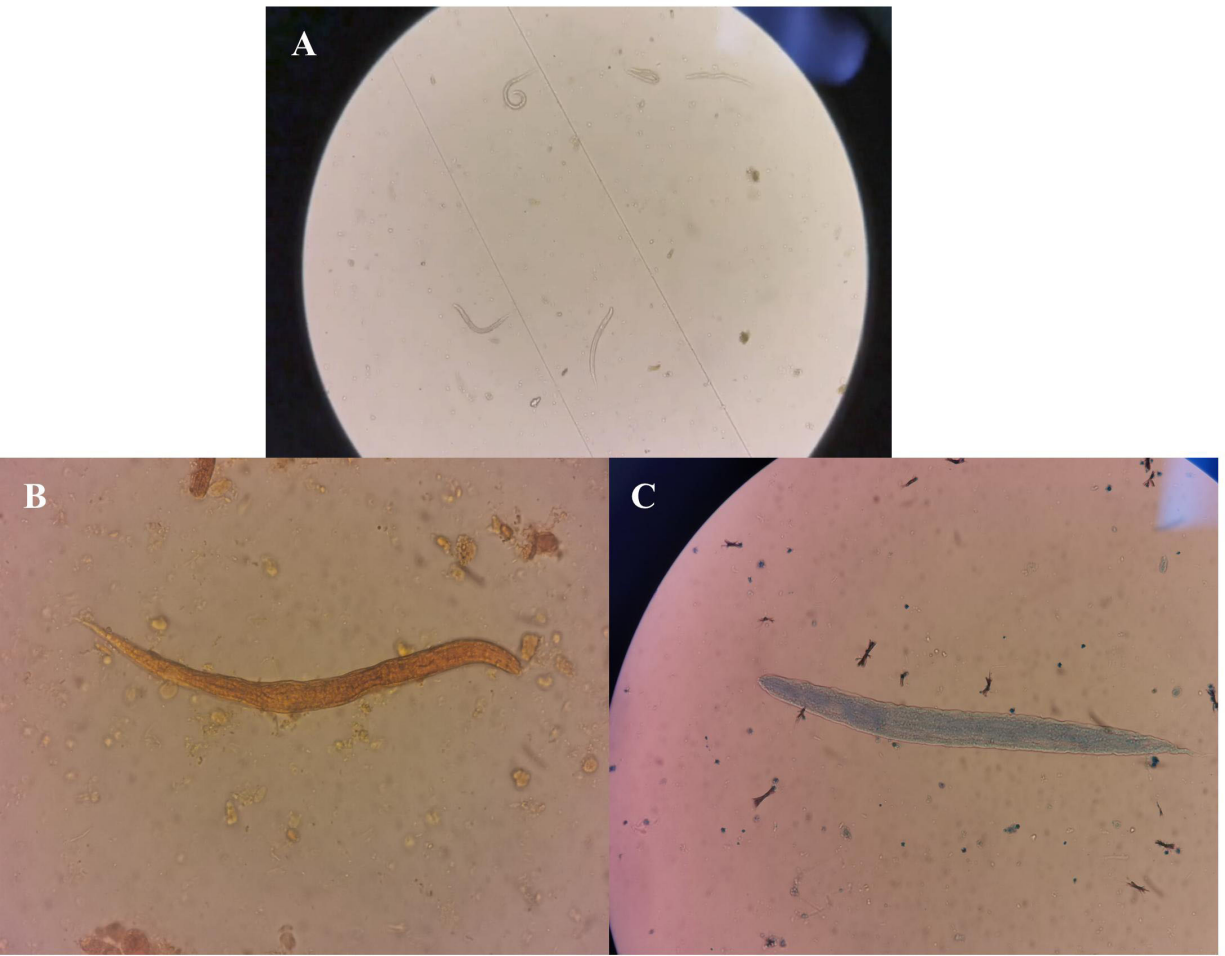
Routine stool test results. **(A)** Active nematodes were detected in the fecal smear. **(B)** Nematodes stained with iodine. **(C)** Reticulocytes stained with fluorescent dye.

A routine stool test carried out on 22 April showed the presence of active nematodes (visible with a microscope) (**Figure 2**), but specific nematode larvae could not be identified at the hospital. On 28 April, the sputum and stool samples were delivered to the Henan Provincial Center for Disease Control and Prevention, and nematode larvae were detected by microscopy in both sputum and stool samples. These results, taken together with the patient’s medical history and mNGS results, confirmed infection with *S. stercoralis*.

**Figure 3:**
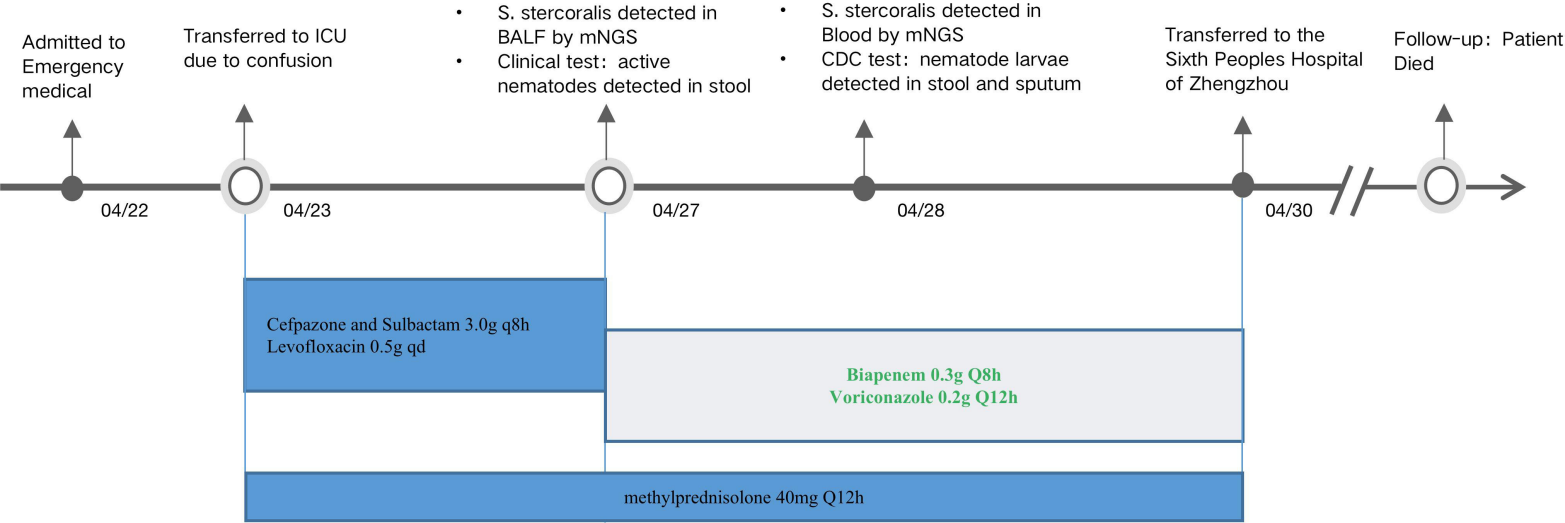
The treatment timeline of the patient.

As no drugs for the specific treatment of *S. stercoralis* infection are available, the patient was first treated with drugs effective against *Klebsiella pneumoniae* and *Aspergillus fumigatus*. Biapenem (0.3 g, q8h) and voriconazole (0.2 g, q12h) were administered, as was methylprednisolone (40 mg, q12h), owing to the patient’s poor immune function. The patient’s temperature dropped after taking the drugs. In accordance with local government requirements for the treatment of infections caused by parasites, the patient was transferred to the Sixth People’s Hospital of Zhengzhou, Zhengzhou, People’s Republic of China, on 30 April. During the follow-up period, because of his long-term low level of immunity and disseminated infection with *S. stercoralis*, the patient died. Their entire treatment process is summarized in **Figure 3**.

## 3 Discussion

*S. stercoralis* is a soil-transmitted nematode that is endemic to tropical and subtropical regions of the world (Nutman, 2017). In the People’s Republic of China, *S. stercoralis* is widely distributed, although it is more common in the southern regions of the country (Wang et al., 2013). The clinical presentation of strongyloidiasis, the disease caused by *S. stercoralis*, is variable in terms of both symptoms and severity. Strongyloidiasis can take the form of an acute infection, chronic persistent infection, or severe disseminated infection, which can involve multiple organs (Ashiri et al., 2019; Bhasin et al., 2020; Oktar et al., 2020). Disseminated disease is characterized by the presence of parasites in organs outside the traditional life cycle sites, such as the skin, lungs, and gastrointestinal tract (Ganesh and Cruz, 2011). It mainly occurs in immunocompromised individuals, including transplant patients, patients taking steroids or immunosuppressive drugs, or those infected with human T-cell lymphophilic viruses (Grover et al., 2011; Kassalik and Mönkemüller, 2011). The clinical presentation of disseminated infections is similar to that of classic strongyloidiasis, and primarily involves nausea, vomiting, diarrhea, weight loss, abdominal pain, gastrointestinal hemorrhage, subacute intestinal obstruction, or eosinophilic granulomatous enterocolitis, which can in turn result in presentations of cough, fever, and dyspnea (Gutierrez et al., 1996; Fardet et al., 2007). Such infections are associated with a mortality rate of 60%–85% in immunocompromised individuals (Asdamongkol et al., 2006).

The patient reported in our study was diagnosed with a disseminated infection caused by *S. stercoralis*, which was detected in sputum, BALF, blood, and stool samples. The patient was an elderly man with a history of bronchitis and intestinal obstruction. Gastrointestinal symptoms were abdominal pain, nausea, intestinal obstruction, and edema. Pulmonary examination revealed the presence of cough, wheezing, dyspnea, and pneumonia, and chest radiographs revealed the presence of confluent lesions, consistent with the symptoms of disseminated infection (Farthing et al., 2020).

In tracing the possible source of infection, we found that the patient’s wife worked as a waste picker and often touched waste cartons from all over the country. Therefore, we hypothesized that, when the patient helped her with cleaning, he may have come into contact with waste from regions where *S. stercoralis* is endemic, and thereby contracted an infection.

After admission, the patient underwent routine clinical tests, for example for stool and urine culture, but the results of these were not definitive; therefore, to identify the pathogen that had caused the infection, blood and BALF samples were tested by mNGS. The results revealed that *Klebsiella pneumoniae*, *Aspergillus fumigatus*, human alphaherpesvirus 1, human betaherpesvirus 5, and *S. stercoralis* were present in the patient’s BALF and blood samples. In addition, *Corynebacterium striatum*, *Enterococcus faecium*, and *Candida albicans* were detected in the BALF sample only; however, these are considered a part of the normal flora in the respiratory tract and opportunistic pathogens of community- and hospital-acquired infections in patients with chronic diseases requiring frequent and prolonged hospitalizations (Renom et al., 2014; Zhou et al., 2020; Talapko et al., 2021). In this case, *Corynebacterium striatum*, *Enterococcus faecium*, and *Candida albicans* were thought to have colonized the respiratory system, given the patient’s clinical symptoms and epidemiology.

In combination with the patient’s symptoms, it was determined that *S. stercoralis* was the main pathogen responsible for the infection. Notably, the use of hormones and prolonged hospital stays may lead to hospital-acquired infections with *Klebsiella pneumoniae* and *Aspergillus fumigatus*. In addition, the patient was immunocompromised for a long period, and this may have been the reason behind the detection of human alphaherpesvirus 1 and human betaherpesvirus 5 in his samples. Given the mNGS results and clinical symptoms, *Klebsiella pneumoniae* and *Aspergillus fumigatus* were first treated with drugs, and, following this, the patient’s temperature decreased. However, the drugs used to treat infections caused by *S. stercoralis* could not be obtained because of a lack of availability brought about by lockdown restrictions during the COVID-19 pandemic. Finally, the patient experienced multisite disseminated infections and eventually died.

Therefore, in the case of individuals who are host to multiple pathogens, it is important that the responsible pathogen is identified as quickly as possible and that targeted drugs are administered. The mNGS technique is highly sensitive and is capable of readily detecting pathogens in samples. However, identifying the actual pathogens responsible for infection based on mNGS reports is rather difficult in clinical settings, as it requires a great deal of clinical, laboratory, and microbiological research experience.

In conclusion, the patient in our study was not treated early and effectively because the pathogen responsible for the infection was not identified in time at the local hospital. After he was admitted to the First Affiliated Hospital of Zhengzhou University, the rare pathogen *S. stercoralis* was detected in the patient’s BALF and blood samples using mNGS. This, in combination with the patient’s medical history and mNGS test results, led eventually to a diagnosis of disseminated infection caused by *S. stercoralis*,. The patient later died as a result of immune deficiency and a lack of available drugs to treat the infection. This case highlights the advantages of mNGS in the detection of rare clinical pathogens and identification of mixed infectious agents, which may serve as an aid for the diagnosis of infectious diseases.

## Data availability statement

The datasets presented in this study can be found in online repositories. The names of the repository/repositories and accession number(s) can be found at https://ngdc.cncb.ac.cn/omix/preview/kcfb85XE OMIX003283.

## Ethics statement

Written informed consent was obtained from the individual(s) for the publication of any potentially identifiable images or data included in this article.

## Author contributions

QX and XX wrote and revised this manuscript. DF, QS, and YS reviewed this manuscript. DF and RD collected the data for this manuscript. All authors contributed to the article and approved the submitted version.
